# Gene-Environment Interaction Research and Transgenic Mouse Models of Alzheimer's Disease

**DOI:** 10.4061/2010/356862

**Published:** 2011-01-10

**Authors:** L. Chouliaras, A. S. R. Sierksma, G. Kenis, J. Prickaerts, M. A. M. Lemmens, I. Brasnjevic, E. L. van Donkelaar, P. Martinez-Martinez, M. Losen, M. H. De Baets, N. Kholod, F. W. van Leeuwen, P. R. Hof, J. van Os, H. W. M. Steinbusch, D. L. A. van den Hove, B. P. F. Rutten

**Affiliations:** ^1^School for Mental Health and Neuroscience (MHeNS), Faculty of Health, Medicine and Life Sciences, European Graduate School of Neuroscience (EURON), Maastricht University Medical Centre, P.O. Box 616, 6200 MD, Maastricht, The Netherlands; ^2^Department of Neuroscience, Mount Sinai School of Medicine, One Gustave L. Levy Place, New York, NY 10029, USA; ^3^Division of Psychological Medicine, Institute of Psychiatry, De Crespigny Park, London SE5 8AF, UK; ^4^Department of Psychiatry, Psychosomatics and Psychotherapy, University of Würzburg, 97080 Würzburg, Germany

In the published version of this paper, the following corrections should be introduced:

1. On the first page, it should be mentioned that “L. Chouliaras and A. S. R. Sierksma are joint-first authors.”
2. At page 11, Section 7 “Electromagnetic field exposure”, line 41: it was incorrectly stated that “…, decreased transthyretin levels have also been found in blood serum of long-term wireless phone.” The correct text should read “…, *increased * transthyretin levels have also been found in blood serum of long-term wireless phone.”

